# Biomimetic hydrogel scaffolds via enzymatic reaction for cartilage tissue engineering

**DOI:** 10.1186/s13104-022-06060-w

**Published:** 2022-05-13

**Authors:** Mehdi Khanmohammadi, Maryam Jalessi, Alimohamad Asghari

**Affiliations:** Skull Base Research Center, The Five Senses Institute, Hazrat Rasoul Akram Hospital, School of Medicine, Iran University of Medical Sciences (IUMS), Tehran, Iran

**Keywords:** Biomimetic hydrogel, Horseradish peroxidase-mediated crosslinking, Cartilage tissue engineering

## Abstract

**Objective:**

We aimed to evaluate cytocompatibility of hyaluronic acid (HA) and gelatin (Gela) conjugation with phenolic groups (Phs) via enzyme-mediated crosslinking. Phenolic moieties were substituted on the backbone of HA (HA-Ph) and Gela (Gela-Ph) and subsequently were subjected for horseradish peroxidase crosslinking in the presence of H_2_O_2_ as an electron donor to create a stable hybrid microenvironment for cellular behavior and cartilage tissue engineering.

**Results:**

Successful synthesis of biopolymers confirmed by NRM and UV–Vis spectrophotometry. The physical characteristic of hydrogels including mechanical properties and water contact angle of hydrogels enhanced with addition of Gela-Ph in HA-based hydrogel. The Gela-Ph showed longest gelation time and highest degradation rate. The cellular studies showed cells did not attach to HA-Ph hydrogel. While, proper cell attachment and proliferation observed on blend hydrogel surface compared with the neat hydrogels which interpret by the existence of cell-adhesive motifs of utilized Gela-Ph in this hydrogel. The encapsulated cells in HA-Ph hydrogel were spheroid and just maintained their viability. Hydrogels containing Gela-Ph, the cells were spindle shape with high degrees of cytoplasmic extension. Overall, the results suggest that hybrid biomimetic hydrogel can provide a superior biological microenvironment for chondrocytes in 3D cartilage tissue engineering.

## Introduction

Articular cartilage with its distinct biomechanical and biochemical characteristics is important for the frictionless movement of articulating joints. Large articular defects are rarely healed even with continuous passive motion [1–5]. Until now, numerous experimental and clinical efforts have been made to induce the healing process within mature articular cartilage with the aim of re-establishing structurally and functionally endurable tissue [1, 2, 6]. Development of novel therapeutic approaches for the acceleration of healing in injured articular cartilage is at the center of attention [7, 8]. Hydrogels with biomimetic features can recapitulate tissue microenvironment biomechanically and biochemically and regulate cellular activity in vitro and in vivo [8, 9]. The ECM-derived compounds such as hyaluronic acid (HA), collagen, gelatin, elastin and glycosaminoglycan are assembled as 3D flexible matrices through various ways of crosslinking reaction [5, 10, 11]. The appropriate method and crosslinking can ensure durability and cellular viability as well as functionality during tissue development [5, 8]. The enzyme**-**mediated hydrogel formation specifically horseradish peroxidase (HRP) crosslinking is proven to be mild and effective when comparing existing physical and chemical approaches [7, 10, 12].

Among biomimetic substrates, HA is recognized as a major ECM component in a variety of tissues especially in load bearing tissues such as cartilage, tendon and bone due to its superior viscoelastic properties [7, 13–15]. The carboxylate functional groups of HA can be chemically modified or phenolated (HA-Ph) to facilitate crosslinking upon enzymatic reaction with tunable properties [13, 14, 16]. Although, the HA-Ph is a promising hydrogel for tissue engineering, the non-adhesive nature limits its application where cell attachment is involved [11, 13, 14]. The addition of gelatin (Gela) as a cell-interactive substrate to the HA-based hydrogel matrices can promote cell adhesion properties of the resultant hydrogels. This component is abundantly present in most of tissues and degrades due to its matrix metalloproteinase sensitive protein sequences. Incorporation of this component provide desirable biomaterial property for in vivo implanting hydrogels [14, 17, 18]. Likewise HA, the Gela structure can be chemically modified for enzymatic crosslinking [10, 14]. Furthermore, the phenolated Gela (Gela-Ph) manipulates the cellular adhesion and spreading due to its cell adhesive ligands. However, similar to collagen based hydrogels, enzyme-crosslinking Gela-based hydrogels are mechanically weak [10, 14, 19].

We expect the composite of HA/Gela hydrogel via HRP-crosslinking reaction would be evaluated for promotion of mechanical and biological characteristics. The potential of subject hydrogel was studied in 2D/3D architectures. Moreover, we have characterized physical properties of hydrogels such as mechanical strength, hydrophobic/hydrophilic and degradation behaviors as necessary factors to explore for cell behavior evaluation.

## Main text

### Materials and method

Sodium HA (MW:1.2 × 10^6^ Da), Gela (Type A, 300 bloom), HRP, *n*-hydroxysuccinimide (NHS), 2-morpholinoethane sulfonic acid (MES), 1-ethyl-3-(3-dimethylaminopropyl) carbodiimide hydrochloride (EDC) and tyramine hydrochloride were obtained from Sigma (Saint Louis, MO, USA).

### Synthesis of phenol conjugated HA and Gela

Biopolymers were synthesized by a general carbodiimide/active ester-mediated coupling reaction according to previous reports [11, 14]. Briefly, HA 1 g and Gela 5 g was gently added to the 100 mL MES solution 50 mM. Afterwards, 0.21 g of NHS, 0.42 g of EDC and 0.25 g of tyramine were sequentially added to each solution and stirred for 20 h. Then HA-Ph was harvested through several sequential precipitation using organic solvents. For Gela-Ph, the remaining chemicals were removed by dialysis against distilled water. Finally, each sample was freeze-dried (Telstar, Spain). The successful synthesis of biopolymers was confirmed through ^1^H NMR and substituted Ph amounts were measured by UV spectrophotometry (Shimadzu RF -5000) at 275 nm.

### Gelation time

Gelation time of polymers were determined according previous reported study [15]. The polymeric solution of 0.72% (w/v) HA-Ph and 3.6% (w/v) Gela-Ph was poured into a 48-well plate at 500 µL/well. Subsequently, the 50 µL HRP 12 unit/mL was added into well and homogenized at 50 rpm. In addition, under mixing, the 12 mM H_2_O_2_ 50 µL was dripped into the well and timing was monitored once the magnetic stirring was halted and the surface of the solution swelled, this time was considered as a hydrogelation period.

### Mechanical and degradation properties of hydrogel

The cylindrical hydrogel prepared for compression test and degradation tests. The compression-repulsion force profiles were measured using the cylindrical shaped hydrogels. The gels were placed in a Table Top Material Tester (EZ Test 500 N) installed with a flat probe of 8 mm in diameter and compressed at a crosshead speed of 2.0 mm/min. To study in vitro degradation, hydrogels were immersed into about 10 mL PBS solution. The samples were then incubated on shaker at 50 rpm and 37 °C. At the indicated time intervals, the samples were taken from the media, washed with the distilled water and weighted. The degradation extent was calculated as the remained mass of sample to its initial mass.

### Cell on hydrogel surface

Freeze dried HA-Ph and Gela-Ph powder were soaked in 80% ethanol for sterilization and then dried using vacuum condition. The sterile samples were dissolved in PBS and poured into a 24-well plates. Then HRP and H_2_O_2_ solutions were respectively added to the well at 10:1:1 volume ratio, respectively. The final concentrations of HA-Ph, Gela-Ph, HRP and H_2_O_2_ were 0.5% (w/v), 3% (w/v), 1 unit/mL and 1 mM respectively. Prepared hydrogels were used for cell-adhesion study by adding culture media containing 5 × 10^4^ ADSCs/wells.

### Cell encapsulation

PBS containing polymer precursor solution and ADSCs at 1.0 × 10^6^ cells/mL was mixed with 1/10 (v/v) of concentrated solutions of HRP and H_2_O_2_, resulting in final concentrations of 1 unit/mL and 1 mM, respectively. Immediately after mixing, 400 µL of the polymeric mixture was poured into 24-well culture plate. The growth profile of encapsulated cells within hydrogels were evaluated via estimation of the mitochondrial activity using a colorimetric assay kit (WST-1) while the amount of water-soluble formazan dye derived from a tetrazolium salt dissolved in DMEM containing the cells showed their growth profiles. Besides, the ADSCs were stained with cell tracking dye kit-green to observe morphology of encapsulated cells in hydrogel using fluorescence microscope.

## Results

**Fig. 1:**
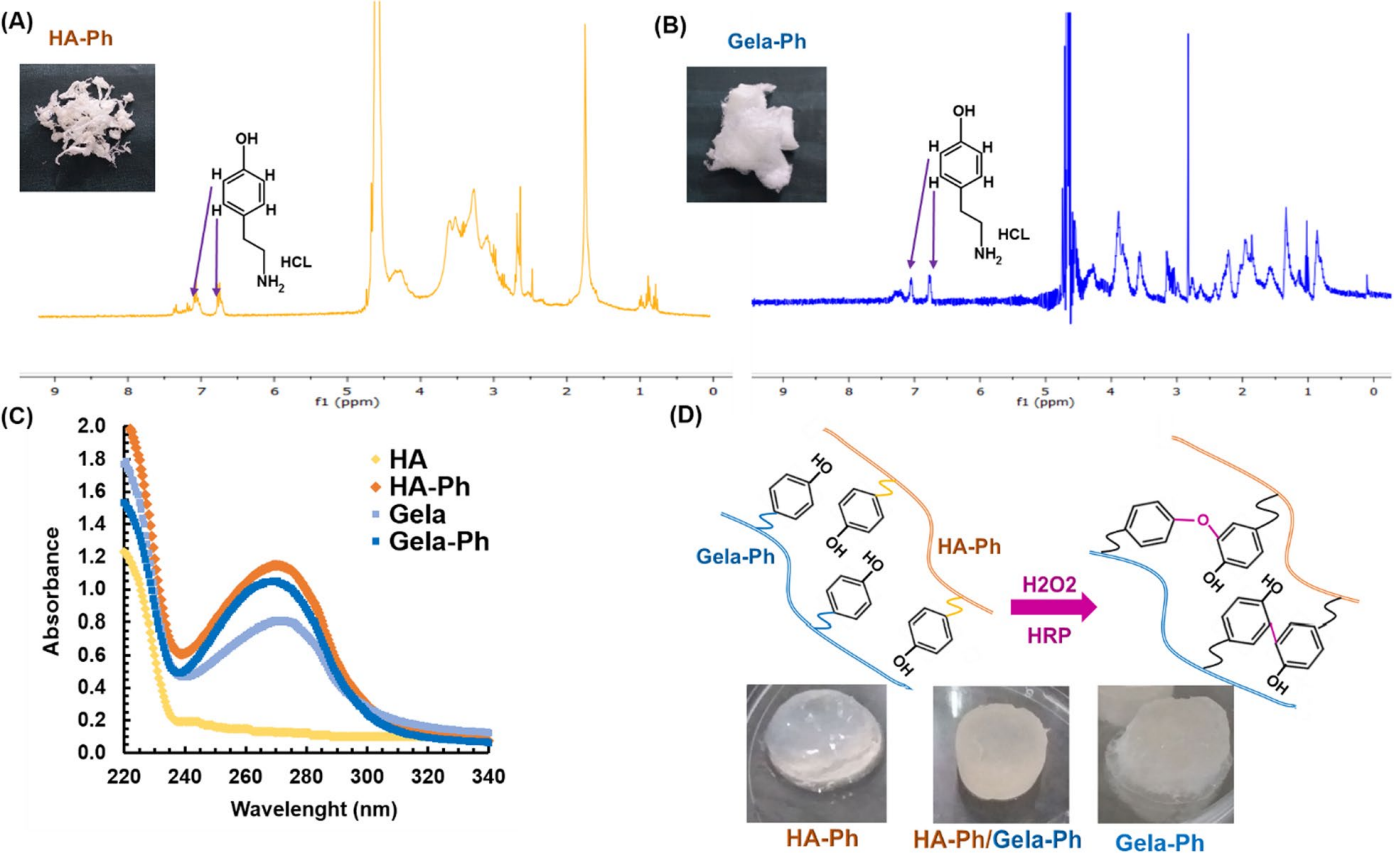
Proton NMR spectra of **A** HA-Ph. **B** Gela-Ph (Ph) in D_2_O. **C** Samples at 0.1% (w/v) dissolved in DI water **D** Schematic of hydrogel formation by an enzymatic catalyzed oxidation reaction and photographs of hydrogels

The conjugation of Ph moieties on resultant polymeric derivatives proved through ^1^H NMR analysis. As shown in Fig. 1A, B, HA-Ph and Gela-Ph NMR spectra were demonstrated appearance of characteristic peaks of Ph groups at 6.8–7.2 ppm chemical shift related to protons in ortho and meta position of conjugated Ph moieties [11, 17]. The degrees of substituted Ph moieties in HA-Ph and Gela-Ph were determined to be 1.81 × 10^–4^ mol-Ph**/**g-HA-Ph and 1.68 × 10^–4^ mol-Ph**/**g-Gela-Ph (Fig. 1C). The hydrogels from synthesized biopolymers prepared through HRP-mediated crosslinking (Fig. 1D).

### Hydrogel characterization

**Fig. 2:**
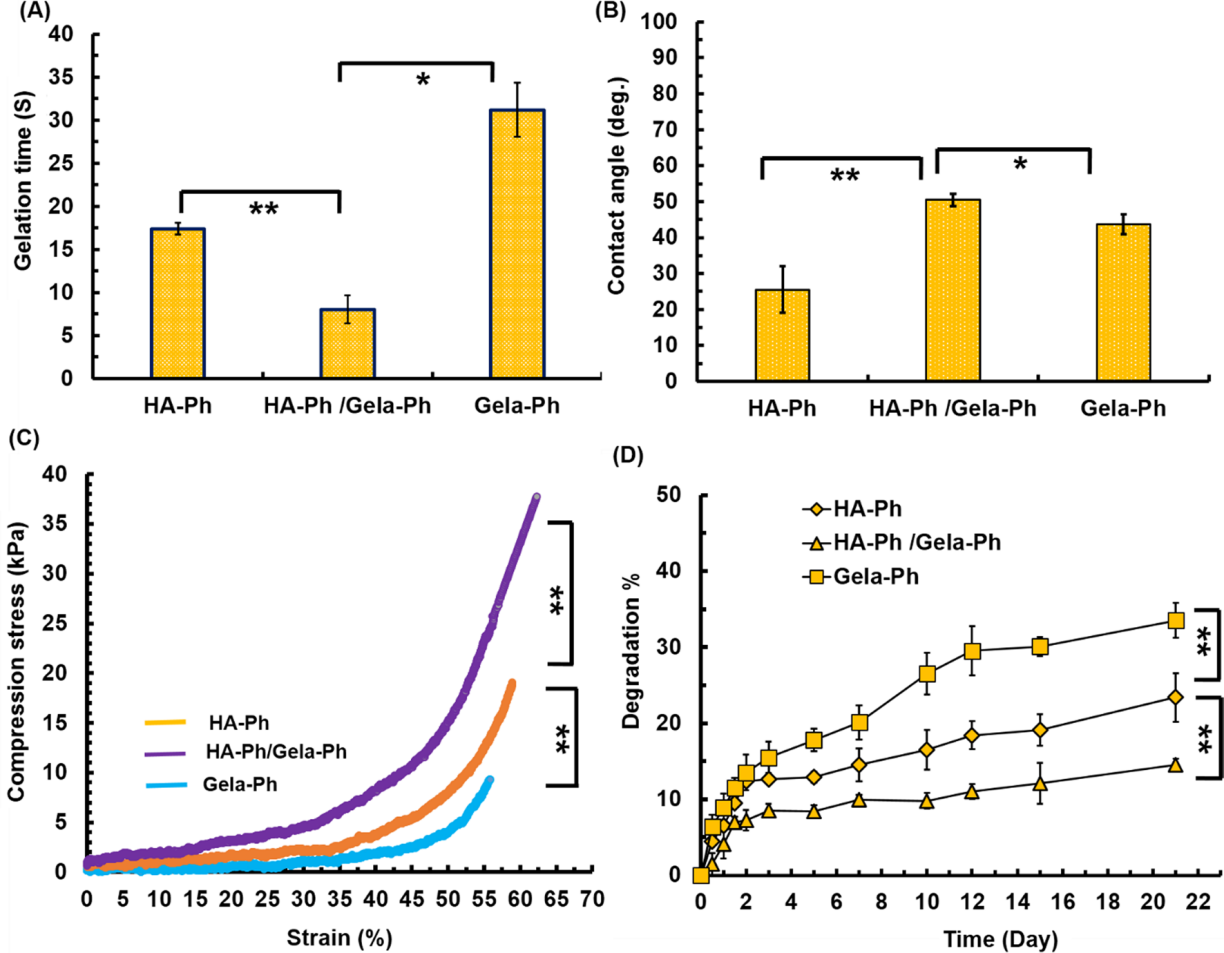
**A** Gelation time. **B** Contact angle. **C** Compression stress. **D** Degradation test. Reported values are shown as the average (n = 4) ± SD (*p < 0.05, **p < 0.01, ns p > 0.05)

The formation of the hydrogels was performed while HRP oxidizes the Ph groups to form free radicals which crosslink the aromatic rings by C–C and C–O coupling using H_2_O_2_ as an electron donor (Fig. 1D). The gelation time of hydrogels varied from few seconds until 35 s. Meanwhile, the Gela-Ph showed longest gelation time at 35 s (Fig. 2A). The gelation time of hybrid HA-Ph/Gela-Ph was shortest among hydrogels which could be due to higher amount of Ph moieties in this condition resulting higher density of crosslinking [16, 20]. The HA-Ph hydrogel demonstrated super-hydrophilic property by contact angle of 22 degree and incorporation of Gela-Ph in HA-Ph increased its hydrophobicity at 52 degrees (Fig. 2B). Regarding mechanical properties of hydrogels, while the lowest stability was related to the Gela-Ph hydrogel and broke at 52% strain with an ultimate compressive strength of 6.5 kPa (Fig. 2C). The Gela-Ph positively influence compressive strength and results showed that the HA-Ph/Gela-Ph hydrogel had the strongest compressive strength with the ultimate compressive strength of 35 kPa and broke at 64.4% strain (Fig. 2C).

One of the critical features of the implantable hydrogels is that the microstructures have to be degraded in a controlled fashion. As is shown in Fig. 2D, the Gela-Ph hydrogel degraded as a function of time until 31%. Degradation of HA-Ph/Gela-Ph was low (about 21%) and became most stable structure.

### Biochemical evaluation of hydrogels

**Fig. 3:**
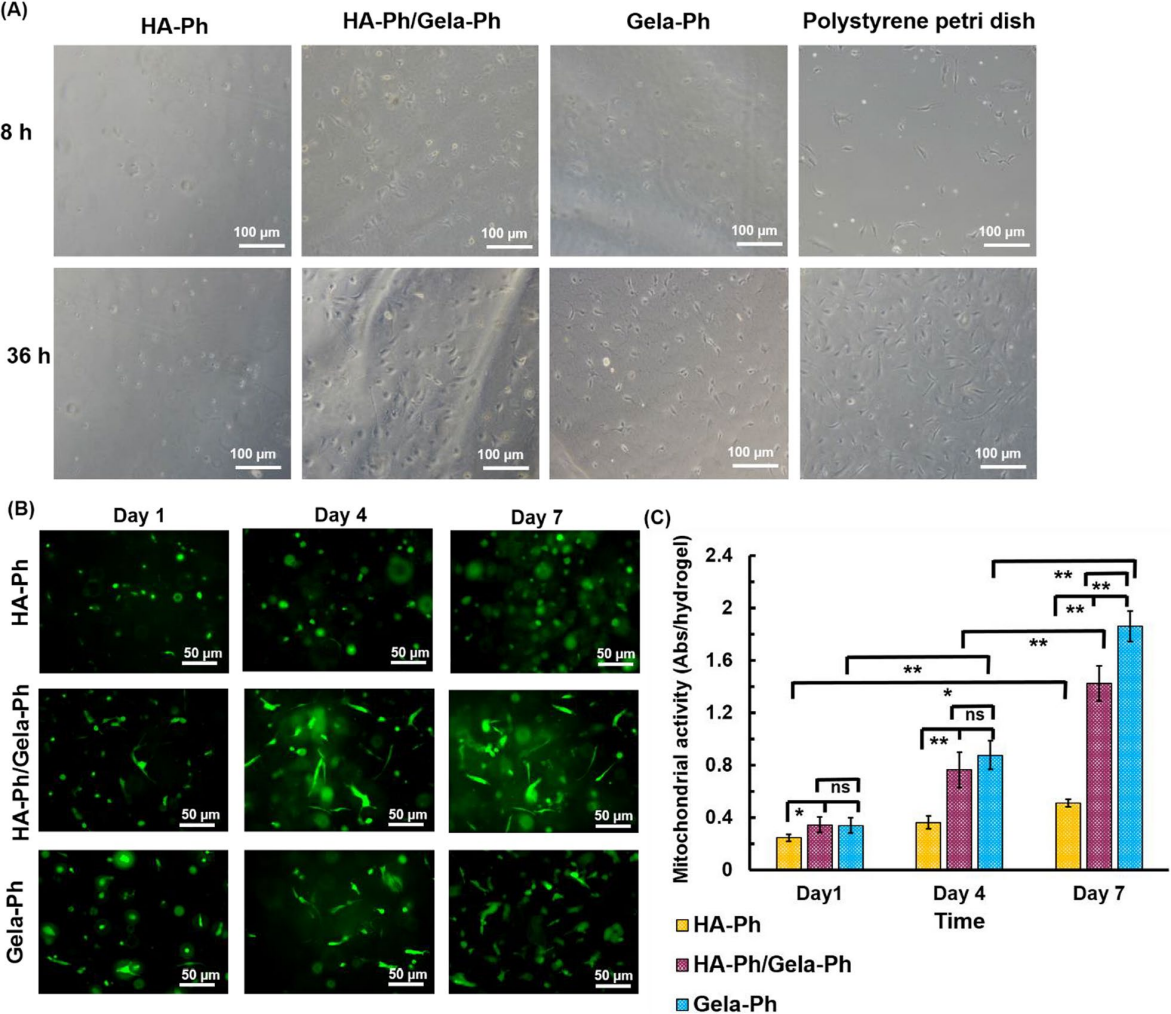
**A** Microphotographs of ADSCs morphology after 8 h and 36 h of seeding on hydrogels. Fluorescence images. **B** and mitochondrial activity **C** of ADSCs encapsulated in hydrogels

The ADSCs totally elongated and spread with reticular network formation on dish culture. Meanwhile, the minor of ADSCs observed on surface of HA-Ph hydrogel and their morphologies were totally in spheroid-shape without any cytoplasmic extension (Fig. 3A). In contrary, the ADSCs attached and elongated in Gela-Ph hydrogel in initial time of culture and showed totally spindle shape by increasing culture time. Interestingly, in HA-Ph/Gela-Ph hydrogel, the ADSCs showed good attachment and spreading similar with Gela-Ph hydrogel (Fig. 3A).

Encapsulated ADSCs did not grow within HA-Ph hydrogel and quantity of mitochondrial activity were remain statistically static without significant change until 7 days (Fig. 3B, C). The ADSCs were grown and proliferated more than 4 times in hydrogel Gela-Ph against to 2.5 times for hydrogel that was made by HA-Ph/Gela-Ph. Nearly the main of cells in HA-Ph were spheroid and could not elongate (Fig. 3C). Likewise, 2D study, hydrogels containing Gela-Ph could observe cell spreading and increase cell elongation affinity. The ADSCs surrounded in non-adhesive HA-Ph hydrogels had no spreading within 3D structure of the gel.

## Discussion

A variety of cell-laden hydrogels have been produced by Ph functionalization of different polymers such as alginate, silk, chitosan, collagen and HA and subsequent peroxidase-mediated crosslinking [5, 10, 20]. Among these biomaterials, the HA and Gela display minor frictional irritation to the surrounding tissues [9, 10, 18]. Also, the crosslinking approach and density within hydrogel matrix may cause the cytotoxicity behavior. Enzymatic crosslinking is a mild approach to induce the formation of 3D hydrogel networks [5, 20]. Here, HA-Ph and Gela-Ph successfully conjugated covalently with Ph groups and hydrogelated via the HRP-catalyzed oxidative crosslinking [21, 22]. The long gelation time of Gela-Ph hydrogel could be interpreted with required time for polymerization of low viscose Gela-Ph aqueous solution and increasing intramolecular network formation and hydrogelation [14]. While, in hybrid condition density of crosslinkable Ph was highest compared with other groups which resulted fastest gelation. The HA-Ph demonstrated superior hydrophilic property and was tuned this property by incorporation of Gela-Ph which would be due to intrinsic properties of Gela-Ph as well as increasing degrees of crosslinking since Gela-Ph hydrogel showed lower hydrophobicity compare with HA-Ph/Gela-Ph hydrogel [7, 23]. The mechanical properties of hydrogel in hybrid condition proved upregulation of hydrogel stability compared with single composed hydrogels. Fabricating cartilage tissue construct is required stable structure to withstand against loading pressure and endured mechanically. Hence, the hybrid condition could be provided proper condition for cartilage tissue engineering due to its mechanical stability and moderate hydrophilicity [4, 8, 9].

The biological features of the prepared biomimetic hydrogels evaluated using hydrogels for 2D and 3D cell culture. As expected, cell morphology was different in the HA-Ph hydrogel with others hydrogels and low number of cells attached in spheroid shape [16, 24]. Cellular morphologies in Gela-Ph incorporated hydrogels were similar with conventional cell culture in spindle shape with filopedia formation which could be reasoned for effectiveness of Gela cell-adhesive motifs. Next, we analyzed the cellular morphology and proliferation ratio of encapsulated ADSCs. Similar with 2D culture for HA-Ph hydrogel, the morphology ADSCs was spheroid in this condition and no cell spreading observed at 7 days. On the other hand, in hybrid hydrogels, the cells were spindle shape and grew properly. In summary, these results recommended that using hybrid hydrogel could upregulate hydrogel specifications as a biomimetic cartilage microenvironment due to improved cellular growth and proliferation as well as biophysical properties of the substrate [4, 7–9].

## Conclusion

Biomimetic hydrogels were obtained using aqueous HA-Ph and Gela-Ph solution containing HRP and H_2_O_2_. The developed hydrogels preserved their structure stability during the culture without specific cytotoxicity. The physical properties of developed hydrogels manipulated and upregulated by alteration of polymeric composition for cell culture system. The potency of prepared hydrogel for cartilage tissue engineering supported by viability of ADSCs in 2D and 3D microenvironment. These results demonstrate that the composite biomimetic hydrogel obtained through HRP-mediated crosslinking have the potential for application in the cartilage tissue engineering.

## Limitation

We showed possibility of HRP-mediated biomimetic hydrogel formation using synthesized HA-Ph and Gela-Ph. However, precise investigation is required for characterization and evaluation of composite HA-Ph/Gela-Ph hydrogel in different concentrations. Also, the quantitative and qualitative analyses on expression of cartilage tissue proteins and gens in evaluating hydrogel conditions are other important requirement to understand proper hydrogel condition for engineering cartilage tissue. It is recommended after optimization of hydrogel condition from physical and biological consistency for cartilage tissue engineering, examine the developed hydrogel for in vivo.

## Data Availability

The data created and analyzed during the current study are available from the corresponding author upon reasonable requests.

## Abbreviations

Hyaluronic acid: HA
Gelatin: Gela
HRP: Horseradish peroxidase
H_2_O_2_: Hydrogen peroxide
EDC: 1-Ethyl-3-(3-dimethylaminopropyl) carbodiimide
NHS: N-hydroxysuccinimide
MES: 2-Morpholinoethanesulfonic acid, monohydrate
NMR: Nuclear magnetic resonance
Ph: Phenol
UV–Vis: Ultraviolet–visible spectroscopy
